# Amygdala connectivity related to subsequent stress responses during the COVID-19 outbreak

**DOI:** 10.3389/fpsyt.2023.999934

**Published:** 2023-02-23

**Authors:** Yuan Zhou, Yuwen He, Yuening Jin, Peter Zeidman, Lianlu Gao, Bei Rong, Huan Huang, Yuan Feng, Jian Cui, Shudong Zhang, Yun Wang, Gang Wang, Yu-Tao Xiang, Huiling Wang

**Affiliations:** ^1^CAS Key Laboratory of Behavioral Science, Institute of Psychology, Beijing, China; ^2^Department of Psychology, University of Chinese Academy of Sciences, Beijing, China; ^3^The National Clinical Research Center for Mental Disorders and Beijing Key Laboratory of Mental Disorders, Beijing Anding Hospital, Capital Medical University, Beijing, China; ^4^Centre for Cognitive and Brain Sciences, University of Macau, Macau, Macao SAR, China; ^5^The Wellcome Centre for Human Neuroimaging, University College London, London, United Kingdom; ^6^Department of Psychiatry, Renmin Hospital of Wuhan University, Wuhan, China; ^7^Unit of Psychiatry, Faculty of Health Sciences, Department of Public Health and Medicinal Administration, Institute of Translational Medicine, University of Macau, Macao, Macao SAR, China; ^8^Hubei Provincial Key Laboratory of Developmentally Originated Disease, Wuhan, China

**Keywords:** amygdala, COVID-19, dorsomedial prefrontal cortex, resting-state functional connectivity, effective connectivity, stress

## Abstract

**Introduction:**

The amygdala plays an important role in stress responses and stress-related psychiatric disorders. It is possible that amygdala connectivity may be a neurobiological vulnerability marker for stress responses or stress-related psychiatric disorders and will be useful to precisely identify the vulnerable individuals before stress happens. However, little is known about the relationship between amygdala connectivity and subsequent stress responses. The current study investigated whether amygdala connectivity measured before experiencing stress is a predisposing neural feature of subsequent stress responses while individuals face an emergent and unexpected event like the COVID-19 outbreak.

**Methods:**

Data collected before the COVID-19 pandemic from an established fMRI cohort who lived in the pandemic center in China (Hubei) during the COVID-19 outbreak were used to investigate the relationship between amygdala connectivity and stress responses during and after the pandemic in 2020. The amygdala connectivity was measured with resting-state functional connectivity (rsFC) and effective connectivity.

**Results:**

We found the rsFC of the right amygdala with the dorsomedial prefrontal cortex (dmPFC) was negatively correlated with the stress responses at the first survey during the COVID-19 outbreak, and the rsFC between the right amygdala and bilateral superior frontal gyri (partially overlapped with the dmPFC) was correlated with SBSC at the second survey. Dynamic causal modeling suggested that the self-connection of the right amygdala was negatively correlated with stress responses during the pandemic.

**Discussion:**

Our findings expand our understanding about the role of amygdala in stress responses and stress-related psychiatric disorders and suggest that amygdala connectivity is a predisposing neural feature of subsequent stress responses.

## 1. Introduction

Psychosocial stressor increases anxiety, depression, and other negative emotions and thus influences individuals’ physical and mental health (1–3). Individual differences in reactions to psychosocial stressors have been observed in some studies (4–6), in which some individuals are more vulnerable to stressors or traumatic events than others. In this context, timely and precise identification of individuals who are vulnerable to stressors is urgent and important. It promotes a more proper allocation of public resources to aid vulnerable individuals, which in turn decreases the likelihood of these potentially vulnerable individuals to develop stress-related psychiatric disorders.

Amounting studies have demonstrated the importance of the amygdala in stress responses and stress-related psychiatric disorders [e.g., post-traumatic stress disorder (PTSD)] (7, 8). For example, previous studies have found abnormal amygdala connectivity in adults with early life stressors (9, 10), relationship between amygdala activity or connectivity with experimentally induced acute stress (11, 12), and altered spontaneous activity or functional connectivity of amygdala in patients with stress-related psychiatric disorders (13–15). These evidences suggest that the amygdala activity or connectivity may be a neurobiological vulnerability marker for stress responses or stress-related psychiatric disorders and will be useful to precisely identify the vulnerable individuals before stressors or traumatic events. However, few studies directly examine this possibility, due to two major difficulties in experimental design. First, the occurrence of natural stressors cannot be foreseen. Second, the acquisition of neuroimaging data before the occurrence of natural stressors is difficult. Two experimental studies have attempted to investigate whether task-induced amygdala activity reflects a vulnerability to trauma exposure (1, 2). These studies reveal that the increased amygdala’s reactivity to stress-related stimuli predicts the subsequent stress symptoms. However, these studies cannot capture the role of amygdala connectivity in predicting real chronic stressors in natural settings.

In December 2019, a mass outbreak of a novel coronavirus infection, named as the novel coronavirus disease (COVID-19), occurred in Wuhan, Hubei province, China. Then, the COVID-19 pandemic affects people around the world. Besides the physical influence due to the infection, the COVID-19 also influences individuals’ mental health (16, 17), not only among the infected patients but also among the general public (18, 19). Thus, the COVID-19 pandemic is taken as an uncertain and unpredictable psychosocial stressor (20). Individual differences in reactions to this stressor have also been observed (21, 22). The current study grasps the unique chance of COVID-19 outbreak and leverages data from an established healthy cohort in Hubei province to investigate whether amygdala connectivity measured before the COVID-19 pandemic is related to subsequent stress responses in the individuals in the geographic pandemic center (i.e., Hubei, China).

Resting-state functional magnetic resonance imaging (fMRI) is a powerful tool to uncover the neural basis of individual differences in human cognitive abilities and behavioral tendencies (23–27). Two studies from one research group have found that the resting-state functional connectivity (rsFC) can predict the feelings of stress or anxiety related to the COVID-19 pandemic (28, 29); however, neither of the studies focused on amygdala connectivity, instead they focused on functional connectome of the whole brain regions. Therefore, it is still unclear whether amygdala connectivity before experiencing stress is a predisposing neural feature of subsequent stress responses while facing an emergent and unexpected event, like the COVID-19 outbreak.

It is noteworthy that these studies applied rsFC to investigate the potential linkage between amygdala connectivity and subsequent stress responses (28, 29). This method, rsFC, which examines correlations between fMRI time series across the brain, does not reveal the causal influence of one neural system on another and thus only describes a non-directed functional interaction (30). Animal studies have demonstrated that the directed interaction between amygdala and other brain regions, such as dorsomedial prefrontal cortex (dmPFC), is highly correlated with the increased anxiety-like behavior in stressed mice (31). Therefore, it is more interesting to investigate whether the causal influences related to the amygdala predict subsequent stress responses in humans.

To capture how the directional interaction between amygdala and brain regions predicts subsequent stress responses in humans, we incorporated the dynamic causal modeling (DCM), a widely adopted framework for effective connectivity analysis (32). DCM can better disclose the causal and directed nature of coupling between intrinsic modes of brain activity (30). This approach has been used to predict individual differences in the cognition of healthy participants and in treatment responses of depressed patients (33, 34). Technologies called *stochastic* and *spectral* dynamic causal modeling (spDCM) are the most recent approaches to characterize effective connectivity during rest (35). Compared to its stochastic counterpart, spDCM, which operates in the frequency domain rather than the time domain, is more computationally efficient and more accurate and sensitive to group differences (36). Therefore, we used the spDCM to estimate effective connectivity and test the hypothesis that the directed connectivity of amygdala or its self-connection, as a predisposing neural feature, is related to subsequent stress responses.

In brief, this study investigates whether resting-state functional and effective connectivity of amygdala measured before the COVID-19 pandemic is related to subsequent stress responses by analyzing data from an established healthy cohort in Hubei, China. Previous studies suggest that the amygdala works together with other brain regions, especially the medial prefrontal cortex (mPFC), to tune the expression of stress-related emotions, such as fear and anxiety (31, 37). Impaired functional interaction between the amygdala and mPFC has been repeatedly reported in both psychiatric patients and animal models and recognized as one of the core neurobiological features across stress-related psychiatric disorders (14, 38–40). Thus, we speculate that the rsFC between amygdala and mPFC measured before the COVID-19 pandemic is related to subsequent stress responses. Then, we furthermore explored how the causal and directed nature of coupling between amygdala and the target region(s) (e.g., mPFC) is related to subsequent stress responses. We are also interested in self-connection within each region. Previous studies have found hyper-responsivity within the amygdala in stress-related psychiatric disorders, such as PTSD (40, 41), and found that amygdala activity is positively correlated with the severity of PTSD symptoms (42). Self-connection in the frame of DCM can be considered as parameterizing the interplay between inhibitory interneurons and pyramidal cells within a brain region, thus reflecting the gain or excitability of neuronal populations (43–45), which is an analogy of activity responsivity during rest. Moreover, we speculate that such relationship between amygdala connectivity and subsequent stress responses will disappear when the stressor weakened. Therefore, we also explored the relationship between amygdala connectivity and stress responses measured after 3 months of the COVID-19 outbreak in Hubei.

## 2. Materials and methods

### 2.1. Participants

**FIGURE 1:**
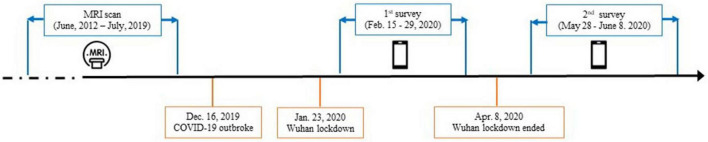
Timeline of data collection with the dates of major events relevant to the COVID-19 pandemic.

Fifty neurologically normal participants were recruited from an established cohort that belongs to an fMRI study conducted at the Renmin Hospital, Wuhan University from June 2012 to July 2019 (46, 47). All participants had reported no history of major psychiatric or neurological illness when they were recruited. Detailed inclusion and exclusion criteria for participants were provided in the original studies (46, 47). All of the participants were invited to complete two surveys, covering from the peak of the outbreak in China in February 2020 to the remission period in June 2020 (Figure 1). Importantly, based on their self-reports, none of the participants were suspected cases or patients with the COVID-19. The first survey was conducted from 15 to 29 February 2020, when residents in Hubei experienced the most serious period of the COVID-19 pandemic. The second survey was conducted 3 months after the first survey (from 28 May to 8 June 2020), when the COVID-19 pandemic had been effectively controlled in China, as indicated by the fact that Wuhan, a Hubei city which was the most seriously affected by the COVID-19, lifted lockdown on 8 April 2020 due to the sharp reduction of daily increased diagnosed cases. Among of these participants, only the data from those participants who lived in Hubei province when they were recruited in the original project, and were living or still lived in Hubei province half a year before the COVID-19 outbreak in Hubei at the time of questionnaire data collection of this study, were used in the current study. These participants (the Hubei Cohort) were assumed to experience a high level of stressors at the first survey.

This study was approved by the Ethics Committee of Renmin Hospital of Wuhan University, and the Institutional Review Board of the Institute of Psychology, Chinese Academy of Sciences. All of the participants gave informed consents online.

### 2.2. Measurements on COVID-19 related stress responses and validation analyses

Following self-report scale development principles (48), we developed a 14-item Stress Behavior Scale (induced by COVID-19) (SBSC) to assess the stress responses specifically related to the COVID-19 pandemic. The sampled behaviors in SBSC are different from the general stress behaviors in existing scales (e.g., Perceived Stress Scale-10, PSS-10). The detailed procedures were included in the Supplementary Material I. SBSC measures the extent to which individuals exhibit several most common COVID-19 induced stress behaviors and feelings. Sample behaviors and feelings in the SBSC include “Repeatedly takes temperature,” “Rushes to buy or hoards daily necessities and food,” “Worries that self or family members would be infected,” and so on. A full list of items is presented in Supplementary Table 1. Participants indicated that the degree to which each of the fourteen items matched their own behaviors and feelings during the COVID-19 pandemic on a six-point Likert scale ranging from 1 = does not match at all to 6 = matches to a great extent. The scale had good internal validity with Cronbach’s alpha amounting to 0.87 and 0.89, respectively for the Hubei Cohort and the non-Hubei Cohort (the validation sample described below). Explorative factor analysis on the combined sample generated one common factor with eigenvalue exceeding 1 and with factor loadings of all items on the common factor exceeding 0.40. We also administered the State-Trait Anxiety Inventory (S-TAI) (49), the PSS-10 (50), and the Patient Health Questionnaire-9 (PHQ-9) (51).

We recruited a group of healthy participants from another established non-clinical cohort conducted in Beijing (*N* = 58, non-Hubei Cohort) (6), administered two surveys during and after the pandemic, and conducted various analyses on the external validity of SBSC. The sample information, survey administration and analytical strategies is provided in Supplementary Material II.

### 2.3. MRI data acquisition and analyses

#### 2.3.1. Imaging protocol

The MRI data were acquired before the COVID-19 pandemic and have been used in previous studies (46, 47). MRI scanning was performed on a 3.0T General Electric Signa HDxt MR scanner in the Department of Radiology, Renmin Hospital of Wuhan University. Resting-state functional images were obtained by using an echo-planar imaging (EPI) sequence [repetition time (TR) = 2,000 ms, echo time (TE) = 30 ms, flip angle = 90°, field of view (FOV) = 220 mm × 220 mm, matrix = 64 × 64, 32 slices, slice thickness = 4 mm, and gap = 0.6 mm) and 240 volumes were obtained. Structural images were collected using a 3D Bravo T1-weighted sequence (TE = 7.8 ms, TR = 3.0 ms, flip angle = 7°, inversion time = 1,100 ms, FOV = 256 mm × 256 mm, matrix = 256 × 256, 188 slices, and voxel size 1 mm × 1 mm × 1 mm). During the resting-state scanning, all participants were instructed to close their eyes and to focus on nothing in particular.

#### 2.3.2. Imaging preprocessing

All imaging data preprocessing procedures were carried out with Data Processing Assistant for Resting-state fMRI version 4.3,^1^ which is based on Statistical Parametric Mapping 12.^2^ The preprocessing procedures include removing first 5 time points, slice-time correction, realignment, co-registration, segmentation for structural images, nuisance covariates regression, normalization to MNI space, and spatial smoothing. The nuisance covariates included the 5 principal components from the individual segmented white matter and the cerebrospinal fluid, 24 motion parameters (6 head motion parameters, 6 head motion parameters one time point before, and the 12 corresponding squared items), and linear and quadratic trends. Particularly, volume-based scrubbing regression by including scrubbing regressors was also included into the multiple linear regression model (52). The time points with a threshold of framewise displacement (FD) >0.5 mm as well as one back and two forward frames were identified and then modeled as a separate regressor in the regression model of the realigned resting fMRI data. After that, the preprocessed images were temporal filtering. For rsFC, a temporal filtering (0.01–0.1 Hz) was conducted. For effective connectivity, a general linear model (GLM) and an F-contrast analysis were used to identify the low frequency fluctuation in effective connectivity analysis based on previous studies (52, 53). Specifically, the voxels showing low frequency fluctuations were identified using a GLM containing a discrete cosine basis set with frequencies ranging from 0.0078 to 0.1 Hz. An F-contrast was specified across the discrete cosine transforms, producing an SPM that identified regions exhibiting BOLD fluctuations within the frequency band.

A gray matter mask was generated by including the voxels in which 90% of participants contained EPI signal and the mean gray matter values were larger than 0.2. All of the following analyses were conducted within this mask.

#### 2.3.3. Functional connectivity analyses

The left and right amygdala derived from the SPM Anatomy toolbox (54, 55) were used as two seed regions for rsFC analysis separately. We calculated the Pearson correlation between the mean time series of the seed and the time series of each voxel within the gray matter mask. After transforming the Pearson correlations into *z*-values, the resulting *z*-valued functional connectivity maps of each seed were entered to the multiple regression analyses to investigate the relationship between amygdala’s functional connectivity and the SBSC score. To remove the confounding effects, we included gender, age, and mean FD as covariates to the regression model. Statistical significance was set at a cluster-defined threshold *p* < 0.001 in conjunction with cluster wise FWE *p* < 0.025 (Bonferroni correction for two seed-based rsFC analyses) to correct for multiple comparisons.

#### 2.3.4. Effective connectivity analyses

According to the results of functional connectivity analyses, spDCM was used to reveal the relationship between directed connectivity and stress responses induced by the COVID-19 pandemic. Specifically, we took the right amygdala and the region identified by functional connectivity analysis (i.e., dmPFC) as the volume of interest (VOI). The principal eigenvariate of the voxels in each VOI was computed separately. Then spDCM analysis was conducted using DCM12.5 implemented in the SPM12 (revision 7497, see text footnote 2). For each participant, a fully connected model was built to investigate whether the stress responses was related to the top-down regulation effect from the dmPFC to the right amygdala, or the down-up regulation effect from the right amygdala to the dmPFC. We were also interested in whether the stress responses were related to the self-connections within each region. Therefore, we constructed a full model consisting of the directed connections between the right amygdala and the dmPFC as well as self-connections within each region.

The inversion of DCM at the first level was performed using spDCM, which fits the complex cross-spectral density using a power-law model of endogenous neuronal fluctuations (35, 36). Then we used Parametric Empirical Bayes (PEB) (56) to model how individual connections relate to group means and individual differences in stress responses related to COVID-19 outbreak indicated by the SBSC scores. Using the Bayesian model comparison implemented in the PEB framework, we compared reduced models that encoded different hypotheses to find the best model, who told us whether there was an effect of SBSC scores on the effective connectivity and, if so, where it was expressed (57, 58). To address this, we performed an automatic search over the reduced PEB models, in which an efficient (greedy) search of the model space was conducted by scoring the evidence for different models (with certain connections switched on or off) based on log model evidence or free energy. In the PEB framework, group-level analysis is conducted using Bayesian inference. It avoids the need to contend with the multiple-comparison problem of classical inference, because the objective is to quantify the posterior probability for effects, rather than determine whether they exceed a significance threshold (59). We computed the Bayesian posterior probability for our effects of interest using variational Bayesian methods, as implemented in the PEB framework. Then, we computed the Bayesian Model Average, which is the average of the parameters from different models weighted by the models’ posterior probabilities, to present the results. Here we focus on effects with posterior probability >0.95, which is considered “strong evidence” for an effect (60). The detailed guidance of conducting these group-level analyses could be found in the tutorials (56, 58).

### 2.4. Follow-up analyses

We repeated the abovementioned functional connectivity analyses to explore the correlations between the rsFC of amygdala and the SBSC scores at the second survey. If the correlation found at the first survey still remained at the second survey, then effective connectivity was furthermore analyzed; otherwise, no more analyses were conducted.

In addition, we explored the relationship between changes in SBSC scores and the rsFC or effective connectivity of amygdala identified in the abovementioned analyses.

## 3. Results

### 3.1. Demographic characteristics and measurements on COVID-19 related stress responses in Hubei-Cohort

Forty-five participants who completed the first survey were recruited in the Hubei Cohort. Among of them, 30 participants also completed the second survey. Table 1 showed the demographic characteristics and measurements on COVID-19 related stress responses in the first and second survey of the Hubei Cohort, and the score differences between the two surveys with paired sample *t*-tests. As shown, after 3 months, when the COVID-19 pandemic had been effectively controlled in China, stress responses measured by the SBSC scores decreased in the Hubei Cohort (26.30 ± 11.43) relative to the first survey (34.73 ± 13.44) in the 30 participants who completed the second survey [*t*(29) = 3.24, *p* = 0.003].

**TABLE 1:** Demographic and behavioral measurements in the main analyses (Hubei Cohort).

	The first survey (*N* = 45) [mean (SD)]	The second survey (*N* = 30) [mean (SD)]	Difference between the first and second survey (*t, p*)
Age (year)	29.38 (4.10)	29.57 (4.51)	
Gender (male/female)	22/23	16/17	
Education (year)	16.67 (4.19)	17.20 (4.44)	
Head motion (mean FD)	0.12 (0.06)	0.12 (0.06)	
PHQ-9	4.16 (3.81)	4.07 (3.71)	*t*(29) = −0.21, *p* = 0.84
TAI	39.98 (8.26)	36.43 (10.01)	*t*(29) = 1.78, *p* = 0.086
SAI	37.58 (11.41)	33.77 (10.43)	*t*(29) = 0.85, *p* = 0.404
PSS	13.58 (4.07)	10.60 (5.97)	*t*(29) = 2.61, *p* = 0.014
SBSC	34.73 (13.44)	26.30 (11.43)	*t*(29) = 3.24, *p* = 0.003

PHQ-9, Patient Health Questionnaire-9; PSS, Perceived Stress Scale; SAI, State Anxiety Inventory; SBSC, Stress Behavior Scale (induced by COVID-19); TAI, Trait Anxiety Inventory.

### 3.2. Validation analyses of SBSC

Sample characteristics of the validation sample (i.e., non-Hubei Cohort) are provided in Supplementary Table 2. Validation analyses of SBSC convergently showed good external validity of SBSC (for details, refer to Supplementary Material III).

### 3.3. Functional connectivity

**FIGURE 2:**
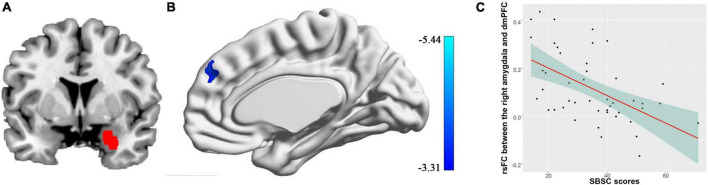
Correlation between rsFC of the right amygdala with the dmPFC and stress responses in a sample consisting of 45 participants who completed the first survey. **(A)** The right amygdala; **(B)** the dmPFC whose rsFC with the right amygdala correlated with the stress behaviors during the COVID-19 outbreak; **(C)** a scatter plot showing the relationship between the rsFC and the stress responses.

We found the connectivity between the right amygdala and the dmPFC (peak coordinates: [8, 48, 36], cluster size = 172 voxels, cluster-level FWE *p* = 0.016) was negatively correlated with the SBSC scores of the first survey, suggesting that the individuals with weaker correlation between the right amygdala and the dmPFC exhibited more stress responses (Figure 2). No significant correlations between the rsFC of the left amygdala and the SBSC scores were found.

### 3.4. Effective connectivity

**FIGURE 3:**
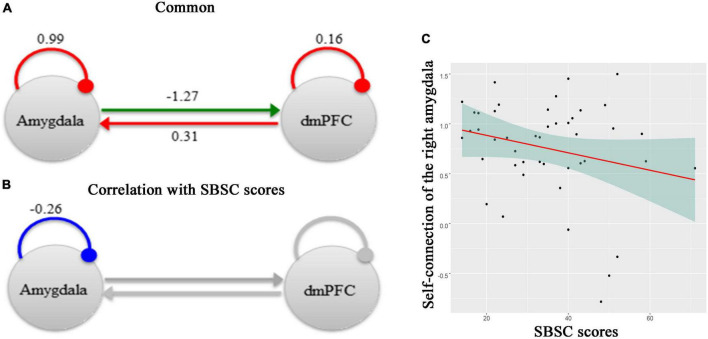
A schematic summarizing effective connectivity between the right amygdala and the dmPFC across participants in the Hubei Cohort and its correlation with stress responses at the first survey. **(A)** The green arrow represents the positive extrinsic effective connectivity and the red arrow represents the negative extrinsic effective connectivity. The parameters are the strength of connectivity. The red arcs represent self-connections. For the self-connections, the parameters are log scaling parameters, which can be converted to units of Hz by: y =–0.5 * exp(x). Where *x* is the log scaling parameter, −0.5 Hz is the prior and y is the self-connection strength in units of Hz. **(B)** The parameter is the effect of the stress responses on the self-connection. All of the other connections in this network, which had no correlations with the SBSC scores, were shown in gray. **(C)** A scatter plot showing the relationship between the self-connection in the right amygdala and the stress responses.

Figure 3 shows effective connectivity between the right amygdala and the dmPFC across all participants in the Hubei Cohort and its correlation with stress responses at the first survey, which exceeded 95% posterior probability based on comparing the approximate log evidence for models with and without each connectivity parameter. In Figure 3A, connectivity parameters are rate constants in Hertz for between-region connections, but to ensure negativity, self-connections are unitless log-scaling parameters that multiply a default value of −0.5 Hz. In terms of DCM for fMRI, a negative value indicates an inhibitory connection, showing that the brain activity of one brain region can decrease the rate of change of activity in another brain region; a positive value indicates an excitatory connection, indicating that the brain activity of one brain region can increase the rate of change of activity in another brain region (32, 56). Thus, we found that there was an inhibitory connectivity from the right amygdala to the dmPFC and an excitatory connectivity from the dmPFC to the right amygdala across participants (Figure 3A). Also, the level of self-inhibition was greater than the expected one under the priors for both regions, as shown by the positive parameter estimates. More importantly, we found a negative effect of the SBSC scores on the inhibitory self-connection of the right amygdala (posterior probability >95%), showing that individuals with weaker self-inhibition of the right amygdala had more stress responses (Figures 3B, C). The parameter in Figure 3B is the effect of the stress responses on the self-connection.

### 3.5. Follow-up analyses

**FIGURE 4:**
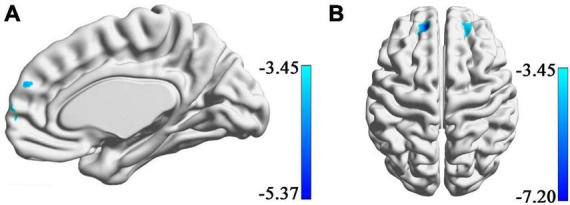
Regions showing correlations between rsFC with amygdala and the stress responses in a sample consisting of 30 participants who completed both of the first and the second surveys. **(A)** A cluster in the dmPFC whose rsFC with the right amygdala correlated with the score of SBSC at the first survey. **(B)** Bilateral superior frontal gyri whose rsFC with the right amygdala correlated with the score of SBSC at the second survey.

Using the data of these 30 participants in the Hubei Cohort, we could still find a negative correlation between the connectivity of the right amygdala with the dmPFC and the SBSC scores of the first survey (uncorrected voxel-wise *p* = 0.001, cluster-wise FWE *p* < 0.008, Figure 4A), which validated our main finding in a smaller sample size. No significant correlations were found between the amygdala’s rsFC and the SBSC scores of the second survey in the Hubei Cohort in the dmPFC. Instead, it was found that the rsFC of the right amygdala with the bilateral superior frontal gyri (SFG) was negatively correlated with SBSC scores at the second survey (uncorrected voxel-wise *p* = 0.001, cluster-wise FWE *p* < 0.025, Figure 4B). Because this finding is contradicting with our hypothesis that such relationship between amygdala connectivity and subsequent stress responses will disappear when the stressor weakened. We had a closer look at the findings. We found that the right SFG identified at the second survey had a small intersection with the dmPFC previously identified at the first survey (Supplementary Figure 1A). And we found a cluster located in the right SFG whose connectivity with the right amygdala was negatively correlated with the SBSC scores at the first survey at a lenient threshold (uncorrected voxel-wise *p* = 0.001, cluster size >50; Supplementary Figure 1B). This cluster was partly overlapped with the right SFG identified at the second survey.

In addition, we found that there was no significant correlation between changes in SBSC scores and amygdala-dmPFC connectivity or self-connection of the amygdala.

## 4. Discussion

This study demonstrated that the amygdala connectivity during rest obtained before the COVID-19 pandemic is related to stress responses during the COVID-19 outbreak in Hubei. Specifically, in an existing cohort of non-clinical population, we found that the functional connectivity between the right amygdala and the dmPFC was negatively correlated with the scores of stress responses at the early stage of the pandemic. Guided by this finding, we further found that the self-connection of the right amygdala was correlated with the scores of stress responses. This suggested that individuals with a weaker self-inhibition (i.e., disinhibition or hyper-activity) of the right amygdala before the pandemic are at a greater risk to exhibit more stress responses during the COVID-19 outbreak.

Previous studies have reported hyperactivity within the amygdala and impaired functional connectivity between the amygdala and mPFC in stress-related psychiatric disorders (14, 31, 37–40) and found that early stress can alter the amygdala’s connectivity (9, 10). Two studies found that amygdala reactivity to tasks is related to vulnerability to trauma-related psychopathology (1, 2). The current study expanded our understanding on the relationship between amygdala and stress in two important ways. First, taking advantage of an existing cohort built before the COVID-19 pandemic, we have the chance to find the evidence that the spontaneous brain activity of amygdala before stress can predict the subsequent stress responses while facing a public health emergency, suggesting that amygdala connectivity is a neural vulnerability factor to a stressful event exposure. Secondly, using effective connectivity, we provide the first evidence for the link between the self-connection in the amygdala and subsequent stress responses.

We found the negative correlation between the amygdala-dmPFC rsFC and the stress responses during the COVID-19 outbreak, suggesting that an individual with weaker amygdala-dmPFC rsFC before stress is more likely to have stress responses during the COVID-19 outbreak. The dmPFC and amygdala are extensively interconnected in the brain and amygdala-dmPFC functional coupling has a major role in fear conditioning and extinction, emotion regulation, and normal and pathological anxiety (14). The dmPFC is important in the conscious appraisal or expression of negative emotion (61), especially in threat appraisal (62). And the dmPFC has been assumed to actively regulate the amygdala through conscious evaluation and appraisal (63). According to a cognitive control model of emotion regulation (64), the neural representation of emotion regulation can be summarized as interactions between prefrontal and cortical system, including the dmPFC, and subcortical systems, especially the amygdala. Along this line, the negative correlation between the amygdala-dmPFC rsFC and the stress responses during the COVID-19 outbreak in our finding suggests that individuals with tight interaction between the dmPFC and the amygdala may have better ability of emotional regulation and thus less stress responses, indicated by the lower SBSC score. This speculation is supported by previous observations in patients with PTSD, who have impaired emotion regulation (65, 66). For example, amygdala-dmPFC connectivity to threat was decreased in patients with PTSD compared to the healthy controls (66) and functional coupling between the amygdala and the dmPFC to unpleasant stimuli was decreased in patients with high level of PTSD compared to those with low level of PTSD symptoms (67). Different from these two studies, we found the negative correlation between the amygdala-dmPFC connectivity and stress responses during rest and extended into a non-clinical population. However, it should be noted that we did not find any significant correlation between the amygdala-mPFC rsFC and trait or state anxiety in our study. Some studies showed that amygdala-mPFC rsFC is positively correlated with trait anxiety (68) or pre-scan anxiety valuation (69). Although the stress responses measured by the SBSC were significantly correlated with trait or state anxiety in this study, the sizes of correlation between SBSC and S-TAI were at small to moderate level, indicating that the stress responses that SBSC captures are somewhat different from state or trait anxiety. Therefore, it is possible that rsFC of amygdala is specifically related to the SBSC in this study.

Using DCM, we found a common model with an inhibitory connectivity from the right amygdala to the dmPFC and an excitatory connectivity from the dmPFC to the right amygdala. Although functional coupling between amygdala and dmPFC has been repeatedly reported, the directionality of the functional interactions between the two regions has only been examined in few studies. During processing of negative emotion, researchers found that the connectivity from the right amygdala to the dmPFC was significant in healthy controls using a method called Granger causality modeling (70, 71). A recent study, which also used the spDCM as we did, found inhibitory connectivity from the right amygdala to the dmPFC and excitatory connectivity from the dmPFC to the right amygdala, as well as the self-connections in both of the two regions, in healthy volunteers during rest in a network including six other regions besides the amygdala and dmPFC (72). Our finding is consistent with these previous studies.

We found the self-connection in the right amygdala was related to the stress responses during the COVID-19 outbreak. In the DCM framework, self-connections are, *a priori*, constrained to be inhibitory (35). This reflects the fact that inhibitory interneurons are restricted to intrinsic anatomical connectivity within the cortex. This means that an increase in self-inhibition corresponds to a reduction in the excitability of neuronal populations to their afferents (and recurrent self-connections) and on the contrary, a decrease in self-connection (i.e., disinhibition) corresponds to an increase in the excitability. Computational accounts under predictive coding interpret these changes in excitability as a failure to attenuate or modulate the precision of prediction errors; namely, the postsynaptic sensitivity of neuronal populations thought to encode prediction errors (e.g., superficial pyramidal cells) (73, 74). In these accounts, the ensuing psychopathology is often related to an imbalance between sensory and prior precision at lower and higher levels in the cortical hierarchy, respectively. The association with reduced self-inhibition (i.e., disinhibition) and the expression of stress-induced behaviors we observed is particularly interesting in light of predictive coding formulations of stress and anxiety (75, 76). The predictive processing formulations of aberrant interceptive inference (77) – in anxiety and stress – often focus on a failure to attenuate the precision of (interoceptive – and related) prediction errors. This results in a hypersensitivity to interoceptive autonomic afferents. Therefore, our findings suggest that individuals with weaker self-inhibition (i.e., disinhibition or hyperexcitability) of the right amygdala would express more stress behaviors, implying the importance of disinhibition/hyperexcitability of the right amygdala in the expression of stress responses. Previous studies have already shown that changes in the local regulation of amygdala excitability underlie behavioral disturbances in stress-related psychiatric disorders or stress responses (e.g., relapse to drug use) (78, 79) and chronic stress causes amygdala output neurons to become hyperexcitable (78, 80–82). However, in these previous studies, the hyperexcitability in the amygdala was observed while facing stressors or after experiencing stress. Our current study extends our knowledge on the role of hyperexcitability (i.e., disinhibition) of the amygdala in stress by finding that individuals with hyperexcitability in the amygdala before facing stressors will show more stress responses. It is possible that the hyperexcitability in the amygdala makes the individuals more vulnerable to uncertainty and unpredictability of environment and thus more likely to take actions while facing stressors.

We did not find the relationship between the amygdala-dmPFC connectivity with the stress responses when the stressor was not so strong, as shown after the stressor weakened, i.e., after 3 months of the COVID-19 outbreak when Wuhan has lifted lockdown. Instead rsFC between the right amygdala and bilateral SFG was found to correlate with SBSC at the second survey. However, it should be noted that there were intersections between the rsFC of the right amygdala correlated with the SBSC scores at the first survey and that at the second survey, which separately located in the right SFG and the dmPFC (Supplementary Figure 1). It indicates that there existed partially consistent rsFC pattern of amygdala correlated with SBSC scores across time. Even though this is a bit contradicting with our hypothesis, it is understandable because the SBSC in Hubei Cohort was significantly reduced at the second survey compared with the first survey, but it was still higher than that in non-Hubei Cohort at the second survey. Thus, the individuals in Hubei Cohort at the second survey still had relatively high stress responses, which might be the reason for relatively stable behavioral correlates of amygdala rsFC across time.

And this relatively stable brain-behavioral relationship may also account for the finding that there was no significant correlation between changes in SBSC scores and amygdala-dmPFC connectivity or self-connection of the amygdala. On the other hand, this negative finding might stem from the fact that stress-related vulnerability and resilience are two correlated but different psychological components that would engage different brain regions (6, 83). In our current study, we aimed to make use of neuroimaging data recorded before the stressors to predict stress response during the earlier stage of the COVID-19 pandemic (i.e., vulnerability). There might be brain connections that could predict the resilience ability to psychosocial stressors, which could be explored in our future studies.

Several limitations should be mentioned. First, the sample size in this study is small because the volunteers were recruited from an established cohort. For the same reason, we cannot find an appropriate control group to conduct formal statistics to test whether the brain-behavior relationship established in the individuals with more stress responses is statistically stronger than those with less stress responses. An ideal control group could be the individuals who took part in the scanning before the pandemic in the same site as the Hubei Cohort but they or their families were not living in Hubei province before the COVID-19 outbreak in Hubei and thus they were presumed free of the pandemic influence (there were only five cases in the current study). Future studies may use established or new cohorts with large sample size and diversified sample pool to validate the current findings. Second, we do not have baseline measures of stress responses or level. Thus, it is not known whether these participants were influenced by other stressors otherwise the COVID-19 pandemic. Third, this study suggests the potential role of amygdala connectivity in predicting stress responses in a non-clinical population. Whether the current findings can be generalized to patients with stress-related psychiatric disorders or other vulnerable populations to stress needs to be explored. Fourth, we validated the construct validity of SBSC with exploratory factor analysis using a combined sample consisting of Hubei and non-Hubei Cohort. We validated the external validity of SBSC with (1) score comparison between Hubei and non-Hubei Cohort, and (2) correlation with S-TAI in the combined sample. A limitation is that we did not additionally recruit another external sample to perform validation with different assessments of reliability and validity. Furthermore, the neurobiological mechanism behind the link between amygdala connectivity and subsequent stress response needs to be explored in future studies by using animal models. Finally, no MRI scanning was conducted during the pandemic, which prevents us from examining the changes in the brain induced by the pandemic stressors.

## 5. Conclusion

In conclusion, our findings support the role of functional coupling between the amygdala and dmPFC in stress responses and provide new evidence that individuals with hyperexcitability (i.e., disinhibition) in the amygdala will be more likely to exhibit stress behaviors while facing stressors. These findings expand our understanding about the role of amygdala in stress responses and stress-related psychiatric disorders and suggest that amygdala connectivity is a predisposing neural feature of subsequent stress responses.

## Data availability statement

The raw data supporting the conclusions of this article will be made available by the authors, without undue reservation.

## Ethics statement

The studies involving human participants were reviewed and approved by the Ethics Committee of Renmin Hospital of Wuhan University and Institutional Review Board of the Institute of Psychology, Chinese Academy of Sciences. Written informed consent for participation was not required for this study in accordance with the national legislation and the institutional requirements.

## Author contributions

YZ: conceptualization, methodology, writing—review and editing, supervision, project administration, and funding acquisition. YH: conceptualization, formal analysis, and writing—original draft. YJ: formal analysis and writing—original draft. PZ and LG: writing—review and editing. BR, HH, and YF: investigation. JC and SZ: formal analysis. YW: investigation. GW: conceptualization and project administration. Y-TX: conceptualization. HW: conceptualization, supervision, project administration, and writing—review and editing. All authors contributed to the article and approved the submitted version.

## Footnotes

1
http://www.restfmri.net

2
http://www.fil.ion.ucl.ac.uk/spm
