# Supplementary material for: Amygdala connectivity related to subsequent stress responses during the COVID-19 outbreak
Source: Front Psychiatry. 2023 Feb 23;14:999934. doi: 10.3389/fpsyt.2023.999934 (PMC9996006; doi:10.3389/fpsyt.2023.999934)
Supplement: Supplementary file 1 [file Data_Sheet_1.pdf]

Supplementary Material for

Amygdala connectivity related to subsequent stress responses during the COVID-19 outbreak

Yuan Zhou[1,2,3*], Yuwen He[1,4], Yuening Jin[1,2], Peter Zeidman[5], Lianlu Gao[1,2], Bei Rong[6], Huan Huang[6], Yuan Feng[3], Jian Cui[3], Shudong Zhang[3], Yun Wang[3], Gang Wang[3], Yutao Xiang[7,4], Huiling Wang[6,8*]

[1] CAS Key Laboratory of Behavioral Science, Institute of Psychology, Beijing 100101, China.

[2] Department of Psychology, University of Chinese Academy of Sciences, Beijing 100049, China.

[3] The National Clinical Research Center for Mental Disorders & Beijing Key Laboratory of Mental Disorders, Beijing Anding Hospital, Capital Medical University, Beijing 100088, China.

[4] Center for Cognition and Brain Sciences, University of Macau, Macao SAR, China.

[5] The Wellcome Centre for Human Neuroimaging, University College London, Queen Square, London WC1N 3AR, UK

[6] Department of Psychiatry, Renmin Hospital of Wuhan University, Wuhan 430060, China.

[7] Unit of Psychiatry, Institute of Translational Medicine, Faculty of Health Sciences, Macao SAR, China.

[8] Hubei Provincial Key Laboratory of Developmentally Originated Disease, Wuhan, China.

Corresponding author:

Yuan Zhou, Ph.D., CAS Key Laboratory of Behavioral Science, Institute of Psychology, 16 Lincui Road, Chaoyang District, Beijing 100101, P.R. China, e-mail: zhouyuan@psych.ac.cn; Huiling Wang, M.D., Department of Psychiatry, Renmin Hospital of Wuhan University, 238 Jiefang Road, Wuhan 430060, China, e-mail: hlwang@whu.edu.cn

This file includes:

Supplementary texts

Table S1

Table S2

Figure S1

References

Supplementary texts

I. The development of a scale assessing COVID-19 related stress responses

A 14-item self-report questionnaire, Stress Behavior Scale (induced by COVID-19) (SBSC), was developed to assess stress responses to the COVID-19 pandemic. Principal investigators conducted interviews to 10 physicians and students from the Renmin Hospital, Wuhan University and Beijing Anding Hospital on the question of "Based on your observation, please list ten most common stress behaviors among general populations during the period of COVID-19". Principal investigators also collected a total number of 50 news with the key words "COVID-19 related stress responses" (in Chinese) on Baidu News (http://news.baidu.com/), one of the most prevalent news search engines in China. Content analyses on interview records and news were performed to synthesize the most frequently mentioned COVID-19 related stress behaviors. Investigators merged similar items to form a final pool of behavior sample, which contained fourteen items. Another three independent raters who were psychological students from University of Chinese Academy of Sciences evaluated whether each behavior in the list was (1) a frequently seen and (2) a typical COVID-19 stress behavior among general populations on the Yes/No option. All students rated 'Yes' on the high frequency and high typicality for all items.

In the questionnaire, participants indicated the degree to which each of the fourteen items matched their behaviors during the COVID-19 pandemic on a six-point Likert Scale ranging from 1=does not match at all to 6=matches to a great extent (Table S1

in the supplementary materials). The scale had good internal validity with Cronbach's Alpha amounting to .87 and .89 respectively for the Hubei Cohort and the non-Hubei Cohort. Explorative factor analysis on the combined sample generated one common factor with eigenvalue exceeding 1 and with factor loadings of all items on the common factor exceeding .40.

In order to test the criteria validity of this scale, we also collected the self-report measurements on anxiety and stress during this survey. Trait anxiety and state anxiety were measured by a Chinese version 40-item scale translated and revised from the State-Trait Anxiety Inventory (S-TAI) (1). The Pearson correlation of SBSC with the Trait Anxiety Inventory (TAI) and the State Anxiety Inventory (SAI) amounted to $r = .45$, $p < .001$, and $r = .24$, $p = .015$ respectively, suggesting the SBSC has good criteria validity. In addition, we used a Chinese version 9-item scale translated from the Patient Health Questionnaire-9 (PHQ-9) (2) to measure depression.

II. Analytical Strategies on the External Validity of SBSC

First, a critical piece of evidence for the external validity of SBSC is that participants in the pandemic center compared to participants in the peripheral area should display higher level of stress responses due to their higher exposure to the pandemic. To test whether participants in the pandemic center may display higher stress responses related to the COVID-19 than those not in the pandemic center, we recruited fifty-eight healthy volunteers (the non-Hubei Cohort) from another established non-clinical pool of an ongoing fMRI study conducted in Beijing (the MRI data are

not used in this study) (3). All of these participants resided outside the Hubei province when they were recruited in the original project and half a year before COVID-19 outbreak in Hubei. Because there were much fewer COVID-19 cases in other provinces outside Hubei province during the first survey (http://www.nhc.gov.cn/xcs/yqtb/list_gzbd.shtml), this cohort was assumed to experience a lower level of pandemic stressors and thus could be useful to validate the questionnaire developed in this study. All of the volunteers in the non-Hubei Cohort were invited to take part in the two surveys as the Hubei Cohort did.

We compared the SBSC scores between the Hubei Cohort and the non-Hubei Cohort at the first survey to test whether the Hubei Cohort experiences higher level of stress responses related to the COVID-19 pandemic in the midst of the outbreak, controlling for potential confounding variables. To further account for the effect of time, we then conducted a repeated-measure ANOVA (without control variables) and a linear mixed effects model (controlling for potential confounding variables) to investigate the main and interactional effects of site (Hubei vs. non-Hubei) and time (the first survey vs. the second survey) on the SBSC scores to compare differences in the level of stress behaviors during and after the outbreak between Hubei and non-Hubei Cohorts.

Second, we calculated the Pearson correlation between SBSC and S-TAI scores among all participants during the first survey at the time of the pandemic outbreak. We expect a moderate correlation between SBSC and S-TAI scores among all participants, as the level of domain-specific COVID-19 stress behaviors should share

some variance with domain-general state and trait anxiety captured by S-TAI.

III. Results of Validation Analysis of SBSC

Table S2 presents the demographic information and COVID-19 related stress responses in the first and second survey of the non-Hubei Cohort, and the score differences between the two surveys with paired sample t-tests.

In order to test the validity of SBSC, we compared the SBSC scores between the Hubei Cohort and the non-Hubei Cohort at the first survey. Before the formal comparisons, we first compared demographic characteristics and other psychological measurements between the two samples. There was no significant difference in education level ($t(101) = -1.82$, $p = .07$), while significant differences in gender composition (*Chi-square* =4.13, $p = .04$) and age ($t(101) = 4.69$, $p < .001$), existed between the Hubei Cohort and non-Hubei Cohort. Higher scores in the TAI ($t(101) = 2.07$, $p = .04$) and SAI ($t(101) = 2.50$, $p = .01$) were also found in the Hubei Cohort and the non-Hubei Cohort at the first survey. There was no significant difference in the level of depression measured by PHQ-9 ($t(101) = 1.09$, $p = .28$) and PSS-10 ($t(98.63) = 1.54$, $p = .13$) between the two groups at the first survey. Regression analyses after controlling for gender (*beta* = .03, $t = .32$, $p = .747$), age (*beta* = -.05, $t = -.50$, $p = .620$), TAI (*beta* = .39, $t = 4.21$. $p < .001$) and SAI (*beta* = -.02, $t = -.25$, $p = .801$) showed that participants in the Hubei Cohort had significantly higher scores in the SBSC than the non-Hubei Cohort at the first survey (*beta* = -.41, $t = -4.31$, $p < .001$), indicating the Hubei cohort was likely to have more stress responses related

to COVID-19 pandemic, and the between-cohort difference in stress responses are not solely due to differences in TAI, SAI or other demographic variables between cohorts.

A repeated-measure ANOVA was conducted to investigate the main effects of site and time and their interaction effect on the SBSC scores. There were significant main effects of site ($F(1,86) = 25.39$, $p < .001$) and time ($F(1,86) = 34.53$, $p < .001$) in the SBSC scores. However, there was no significant site × time interaction effect ($F(1,86) = 0.02$, $p = .89$). As Hubei Cohort showed higher TAI and SAI than non-Hubei Cohort and they differed in gender and age compositions, we controlled for gender, age, TAI and SAI in the linear mixed effects model and reexamined the main and interactional effect of site and time on SBSC. We still found significant main effects of site ($F(1,92.49) = 26.85$, $p < .001$), time ($F(1,89.70) = 34.77$, $p < .001$) in the SBSC scores, and non-significant site × time interaction effect ($F(1,89.70) = .35$, $p = .558$). These findings suggest the participants in the Hubei Cohort remained higher stress responses across the outbreak and remission of the pandemic than those in the non-Hubei Cohort. All of these analyses indicated the good criterion validity of SBSC.

We then computed the correlations between the SBSC and TAI and SAI. We found that SBSC had significant correlations with both TAI ($r = .45$, $p < .001$) and SAI ($r = .24$, $p = .015$) among all participants in the first survey.

Table S1. The scale items of SBSC

Num.	Items
1	Repeatedly takes temperature.
2	Frequently washes hands; the frequency or the amount of time on handwashing is higher than past.
3	Thoroughly disinfects the house everyday
4	Afraid that the current mask storage is insufficient and the masks lack protective capability.
5	Wears goggles or other eye protection equipment when going outdoors.
6	Wears raincoats or other protective clothing when going outdoors.
7	Often feels tired and uncomfortable.
8	Rushes to buy or hoards daily necessities and food
9	Afraid of going outdoors, even if there is a severe food shortage.
10	Worries that self or family members would be infected.
11	Gets nervous when thinking about the harm of COVID-19.
12	Spends considerable amount of time every day reading about news related to COVID-19.
13	Calls mental support hotlines or seeks for online metal counseling services.
14	Suspects oneself of being infected by COVID-19.

Table S2 Demographic and behavioral measurements in the validation analysis of SBSC for the non-Hubei Cohort

	The first survey (non-Hubei) *(N=58)* (*Mean (SD)*)	The second survey (non-Hubei) *(N=58)* (*Mean (SD)*)	Difference between the first and second survey (*t*, *p*)
Age (year)	25.48 (3.50)	25.48 (3.50)	
Gender (male/female)	17/41	17/41	
Education (year)	17.97 (2.60)	17.97 (2.60)	
PHQ-9	3.26 (4.36)	2.45 (3.88)	$t(57) = 1.80$, $p = .078$
TAI	36.17 (9.96)	35.12 (11.11)	$t(57) = 1.03$, $p = .305$
SAI	31.98 (11.11)	31.88 (11.49)	$t(57) = .10$, $p = .924$
PSS	12.02 (6.17)	11.90 (6.34)	$t(57) = .14$, $p = .889$
SBSC	22.93 (9.76)	17.17 (5.78)	$t(57) = 4.68$, $p < .001$

Abbreviations: PHQ-9, Patient Health Questionnaire-9; PSS, Perceived Stress Scale; SAI, State Anxiety Inventory; SBSC, Stress Behavior Scale (induced by COVID-19); TAI, Trait Anxiety Inventory.

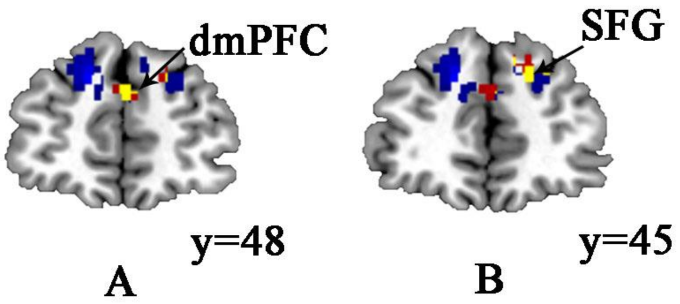

Figure S1 The rsFC of the right amygdala correlated negatively with SBSC. A: The rsFC of the right amygdala correlated negatively with the SBSC at the second survey in the Hubei cohort (uncorrected voxel-wise p=0.001, cluster-wise FWE $p < 0.025$, shown in blue), including a sub-cluster in dmPFC (extending to SFG to form a cluster), which overlapped with the dmPFC identified at the first survey. The rsFC identified at the first survey is shown in red and the overlapping brain area is marked as yellow. B: The rsFC of the right amygdala correlated negatively with the SBSC at the first survey in the Hubei cohort at a lenient threshold (uncorrected voxel-wise p=0.001, cluster size > 50, shown in red), including a cluster in the right SFG overlapped with that identified at the second survey. The rsFC identified at the second survey is shown in blue and the overlapped region is marked as yellow.

References:

1. Spielberger C. State-Trait Anxiety Inventory: Bibliography (2nd, Editio ed.). Palo Alto: Consulting Psychologists Press; 1989.
2. Kroenke K, Spitzer RL, Williams JB. The PHQ-9: validity of a brief depression severity measure. Journal of general internal medicine. 2001;16(9):606-13.
3. Zhang SD, Cui J, Zhang ZF, Wang Y, Liu R, Chen XY, et al. Functional connectivity of amygdala subregions predicts vulnerability to depression following the COVID-19 pandemic. Journal of Affective Disorders. 2022;297:421-9.
